# Water Pipes

**Published:** 1871-08

**Authors:** 


### WATER PIPES.

Wooden water pipes are the only certainly safe and healthful yet known, and, where it is possible, the wood pipe should deliver the water into the dwelling.

A great deal has been said and written about one metal being lined with another, but as no two metals are of equal expansibility, there is liability to crack, and wherever a crack is, there will be a corrosion, a decomposition, more or less hurtful, saying nothing of a galvanic process which takes place between most metals in water.

No water from spring or well, or stream or lake, is perfectly pure; consequently there must be a greater or less chemical effect going on all the time where the water is in contact with the metal. Glass tubes are indestructible by any ordinary waters, but they are not joinable.

Iron pipes will rust, but the rust of iron is least injurious to health. Lead is the favorite material, because it is cheap and flexible; some waters passing through it will poison the family, others will not; and as the waters of all localities differ in their chemical constituents, no one cares to experiment on their own families to determine whether the water supplied to a particular house by lead pipes is poisonous or not.

In Edinburgh, a large percentage of lead is found in the water used, but it has not proved appreciably unhealthful to the city; perhaps there are other elements in the water there which antagonize the ill effects of the lead pipes.

It is a fact observed in several localities, that the water dissolves the lead to a certain extent, and beyond that point there is no further corrosion or oxidation, or but very little. Pure water, distilled water, acts so powerfully on lead as to become very poisonous, but it is not so strong in its action on zinc. Very lately a new house near Boston was piped with iron pipes coated with another metal: a member of the family soon died, and on examination of the body, decomposed zinc was found in the stomach; the whole family sickened. In large towns and cities, where water is incessantly running along the pipes day and night, lead and iron pipes are ordinarily safe enough; but in detached, single houses, they are never safe. No one is justifiable in introducing water into a new house, in a new locality, in any other way than through wooden or glass pipes. The risk is too great.

